# Notes on Preparations for the Sick

**Published:** 1889-09

**Authors:** 


### Notes on Preparations for the Sick.

**La Bourboule Water.**—INGRAM & ROYLE, London.

The mineral waters of Auvergne have long been celebrated, and the number of visitors who annually go to Vichy, Royat, Le Mont Dore, and La Bourboule is steadily increasing. A visit to the health-resorts of the Puy-de-Dôme, and residence at an altitude of three or four thousand feet amongst the mountains, are necessary adjuncts to a due utilisation of the mineral waters at their source. Doubtless this is the most satisfactory method; no matter how valuable the water by itself may be, the adjuncts provided for invalids at the source are an important element in "the cure." It has sometimes been questioned whether the water itself might not be dispensed with, and the cure effected by means of the adjuncts only; but in the case of the waters of La Bourboule no such criticism will bear investigation. The composition of the water at once indicates its applicability to the treatment of a large proportion of diseases of the skin, and various forms of scrofulous disease: the fact that it contains so large a proportion of arseniate of soda as nearly two grains per gallon puts adverse criticism aside, and indicates its utility in all those diseases where arsenic is in steadily increasing use and has a well-established reputation. La Bourboule water contains the largest proportion of arsenic known to exist in any natural water, and it is combined with various salines such as exist in normal blood. The combination is found by experience to be superior to any pharmaceutical preparation: in this form the arsenic appears to be more efficacious in smaller dose, and less likely to irritate the digestive organs. The dose varies from half a tumblerful to two or three in the day; it may be taken at meal-times, cold or warm, either by itself, or mixed with wine or milk. The duration of the course at La Bourboule is twenty-one days; but it not infrequently happens that the system becomes saturated in a shorter time than this.

---

**Elect Extract of Cocoa.**—H. J. ROWNTREE & Co., York.

"The 'Elect' Cocoa is characterised by extraordinary strength and by a refreshing and delightful taste and aroma."

Its characteristics are purity, strength, and flavour. It is highly spoken of by those who have examined its chemistry, and we doubt not is all that it pledges itself to be.

There are so many good preparations of cocoa in the market, that it would be difficult to say which is the best. Some say one, some say the other: those who like cocoa should at least try this one.

---

**Milk-Preparations.**—The Swiss Milk Co., St. Gall, Switzerland.

We have received some of the preparations of this Company.

The **Pure Compressed Milk Extract** is a milk in powder, and is prepared from fresh cow's milk and guaranteed not to contain any foreign or preservative matter. It is composed solely of dried-up milk-products, and being free from water possesses the advantages of little bulk and light weight. It dissolves readily in hot water, and yields a product equal to the best cow's milk.

A **Sweetened Extract of Milk** is made for those who prefer a mixture of the milk-powder with refined cane-sugar.

These powders can be kept indefinitely, even when exposed to the air, without undergoing change, and are therefore very convenient for travelling and exportation.

The same firm also sends us samples of **Dr. Otto Krueger's Chocolate Milk** in powder, which is prepared also with the addition of **Legumin** or of **Malto-legumin.** It is also issued in the form of tablets.

All these preparations have the milk-powder as their basis. The combination with chocolate and with malto-legumin must have a high nutritive value; they are excellent articles of food both in health and disease, are small in bulk, and easily prepared for use.

---

**Lanoline Cream.**—T. Buxton, Bristol.

The advantages of lanoline as a basis for many ointments are now well known: its stickiness is the only disadvantage, and this can easily be removed by admixture with vaseline or vaseline oil. Some such combination as this, with a considerable percentage of water, constitutes the basis of many proprietary articles now much vaunted. The lanoline cream sent to us by Mr. Buxton forms an excellent ointment, and may be used as the basis for a great variety of combinations.

